# Supplementary material for: Low Preoperative Mean Platelet Volume/Platelet Count Ratio Indicates Worse Prognosis in Non-Metastatic Renal Cell Carcinoma
Source: J Clin Med. 2021 Aug 19;10(16):3676. doi: 10.3390/jcm10163676 (PMC8396988; doi:10.3390/jcm10163676)
Supplement: Supplementary file 1 [file jcm-10-03676-s001.zip › jcm-1321914-supplementary.pdf]

Journal of Clinical Medicine

Supplementary materials

Low Preoperative Mean Platelet Volume/Platelet Count Ratio Indicates Worse Prognosis in Non-Metastatic Renal Cell Carcinoma

Yu-Chiao Lin [1,†], Hau-Chern Jan [1,2,†], Horng-Yih Ou [3,4], Chien-Hui Ou [1,*] and Che-Yuan Hu [1,3,*]

1 Department of Urology, National Cheng Kung University Hospital, College of Medicine, National Cheng Kung University, Tainan 704, Taiwan Tainan, Taiwan; whatslin@gmail.com (Y.-C.L.); jan.hauchern@gmail.com (H.-C.J.)
2 Division of Urology, Department of Surgery, National Cheng Kung University Hospital Dou-Liou Branch, Yunlin 640, Taiwan
3 Institute of Clinical Medicine, College of Medicine, National Cheng Kung University, Tainan 704, Taiwan; wahoryi@mail.ncku.edu.tw
4 Division of Endocrinology and Metabolism, Department of Internal Medicine, National Cheng Kung University Hospital, College of Medicine, National Cheng Kung University, Tainan 704, Taiwan
* Correspondence: donou1969@yahoo.com.tw (C.-H.O.); greatoldhu@gmail.com (C.-Y.H.)
† These authors contributed equally to this work.

Table S1. The sensitivity test to confirm MPV/PC as an unfavorable prognostic factor.

	Progression free survival			
	Univariate analysis		Multivariate analysis	
	Hazard ratio	P value	Hazard ratio	P value
**MPV/PC ratio** Low vs High (After excluding high ECOG PS, remaining N = 86)	6.453 (1.388–29.987)	0.017*	5.556 (1.121-27.528)	0.036*
**MPV/PC ratio** Low vs High (After excluding CKD stage V, remaining N = 84)	6.673 (1.458–30.542)	0.014*	4.977 (1.012-24.473)	0.048*

Factors included in multivariate analysis were pathological stage, MPV/PC, tumor necrosis and tumor grade.
